# Detailed volumetric analysis of the hypothalamus in behavioral variant frontotemporal dementia

**DOI:** 10.1007/s00415-015-7885-2

**Published:** 2015-09-04

**Authors:** Martina Bocchetta, Elizabeth Gordon, Emily Manning, Josephine Barnes, David M. Cash, Miklos Espak, David L. Thomas, Marc Modat, Martin N. Rossor, Jason D. Warren, Sebastien Ourselin, Giovanni B. Frisoni, Jonathan D. Rohrer

**Affiliations:** Dementia Research Centre, Department of Neurodegenerative Disease, UCL Institute of Neurology, 8–11 Queen Square, London, WC1N 3BG UK; Laboratory of Alzheimer’s Neuroimaging and Epidemiology, IRCCS Istituto Centro San Giovanni di Dio Fatebenefratelli, Brescia, Italy; Department of Molecular and Translational Medicine, University of Brescia, Brescia, Italy; Translational Imaging Group, Centre for Medical Image Computing, University College London, London, UK; Memory Clinic and Laboratory of Neuroimaging of Aging, University Hospitals and University of Geneva, Geneva, Switzerland

**Keywords:** Hypothalamus, Eating disorders, Frontotemporal dementia, Volumetric MRI

## Abstract

Abnormal eating behaviors are frequently reported in behavioral variant frontotemporal dementia (bvFTD). The hypothalamus is the regulatory center for feeding and satiety but its involvement in bvFTD has not been fully clarified, partly due to its difficult identification on MR images. We measured hypothalamic volume in 18 patients with bvFTD (including 9 *MAPT* and 6 *C9orf72* mutation carriers) and 18 cognitively normal controls using a novel optimized multimodal segmentation protocol, combining 3D T1 and T2-weighted 3T MRIs (intrarater intraclass correlation coefficients ≥0.93). The whole hypothalamus was subsequently segmented into five subunits: the anterior (superior and inferior), tuberal (superior and inferior), and posterior regions. The presence of abnormal eating behavior was assessed with the revised version of the Cambridge Behavioural Inventory (CBI-R). The bvFTD group showed a 17 % lower hypothalamic volume compared with controls (*p* < 0.001): mean 783 (standard deviation 113) versus 944 (73) mm^3^ (corrected for total intracranial volume). In the hypothalamic subunit analysis, the superior parts of the anterior and tuberal regions and the posterior region were significantly smaller in the bvFTD group compared with controls. There was a trend for a smaller hypothalamic volume, particularly in the superior tuberal region, in those with severe eating disturbance scores on the CBI-R. Differences were seen between the two genetic subgroups with significantly smaller volumes in the *MAPT* but not the *C9orf72* group compared with controls. In summary, bvFTD patients had lower hypothalamic volumes compared with controls. Different genetic mutations may have a differential impact on the hypothalamus.

**Electronic supplementary material:**

The online version of this article (doi:10.1007/s00415-015-7885-2) contains supplementary material, which is available to authorized users.

## Introduction

Behavioral variant frontotemporal dementia (bvFTD) is a neurodegenerative disorder characterized by atrophy in the frontal and temporal lobes and progressive behavioral and cognitive impairment [1]. Although the majority of cases are sporadic, about 10–20 % are due to an autosomal dominant mutation in one of three genes: microtubule-associated protein tau (*MAPT*), progranulin (*GRN*), and chromosome 9 open reading frame 72 (*C9orf72*) [2]. One of the characteristic symptoms of bvFTD is the development of abnormal eating behaviors such as hyperphagia, changes in food preference, and craving for sweet foods, which are found in the majority of patients [3–5], and have been shown to help discriminate bvFTD from Alzheimer’s disease [6, 7]. However, the neuroanatomical correlates of abnormal eating behavior in bvFTD are unclear. Previous studies have suggested the importance of an orbitofrontal–insular–striatal brain network [8, 9] but one study has also investigated the role of the hypothalamus, finding a correlation of abnormal eating behavior with lower volumes of the posterior hypothalamus [10].

The hypothalamus is the regulatory center for feeding and satiety [11]. It is composed of a number of different subnuclei and is highly interconnected with other parts of the central nervous system, particularly the brainstem, limbic system, and cortex [12, 13]. However, the hypothalamus is difficult to identify on magnetic resonance imaging and a detailed anatomical analysis of subdivisions of the hypothalamus has not yet been performed in bvFTD. In this study, we aimed to develop an optimized manual segmentation of the hypothalamus and its subunits using a novel protocol, and then use this to investigate patterns of atrophy in bvFTD and specifically whether differences could be seen in different genetic mutations.

## Methods

### Participants

Eighteen subjects fulfilling the criteria for the diagnosis of bvFTD [1] were recruited consecutively from a tertiary referral cognitive disorders clinic at the National Hospital for Neurology and Neurosurgery, London, UK. Nine subjects carried a mutation in the *MAPT* gene and 6 carried a pathogenic expansion in the *C9orf72* gene. The other three bvFTD subjects tested negative for mutations in *MAPT, C9orf72*, and *GRN*. Eighteen healthy controls were also recruited. Subjects’ characteristics are summarized in Table 1. Written informed consent was obtained from all patients and controls, and the local ethics committee approved the study. Each patient underwent a standardized history and neurological examination (including assessment of function using the Frontotemporal dementia Rating Scale [14] ), neuropsychometry (including the MMSE), and assessment of behavioral symptoms using the Cambridge Behavioural Inventory Revised version (CBI-R) [15]. A subset of four questions on the CBI-R addresses the frequency of abnormal eating behavior scoring 0 for never occurring, 1 occurring a few times per month, 2 occurring a few times per week, 3 occurring daily, and 4 occurring constantly. The questions ask about whether sweet foods are preferred, whether the subject wants to eat the same foods repeatedly, whether their appetite is greater than before and whether there has been a decline in table manners. The total score for abnormal eating behavior was converted into percentage of impairment using methods described previously, where 1–50 % is classified as mild or moderate, and 51–100 % is classified as severe or very severe [16].

**Table 1:** Demographic, clinical, and behavioral variables for the bvFTD patients and controls

	Controls	bvFTD	*MAPT* subgroup	*C9orf72* subgroup
Number of subjects	18	18	9	6
Gender, male	9 (50 %)	15 (83.3 %)	7 (77.8 %)	5 (83.3 %)
Age at scan (years)	56.4 (14.3)	63.3 (9.1)	59.5 (9.0)	65.1 (7.2)
Disease duration (years)	N/A	9.1 (5.5)	8.0 (5.6)	10.8 (6.4)
FRS (/100)	N/A	33 (24)	38 (26)	28 (25)
		Range 3–73	Range 7–73	Range 3–67
Age at onset (years)	N/A	54.3 (8.5)	51.4 (6.3)	54.3 (9.8)
Education (years)	14.2 (3.0)	14.3 (4.3)	14.2 (4.8)	13.3 (3.9)
MMSE (/30)	29.2 (1.2)	25.0 (4.4)*	25.8 (5.0)	24.0 (4.0)*
CBI-R Total (/180)	N/A	76.5 (31.8)	76.4 (36.9)	78.7 (33.4)
CBI-R eating disturbance score (/16)	N/A	7.7 (3.9)	7.9 (4.2)	8.3 (3.2)
CBI-R: “prefers sweet foods more than before” (/4)	N/A	2.5 (1.4)	2.8 (1.6)	2.3 (0.8)
CBI-R: “wants to eat the same foods repeatedly” (/4)	N/A	2.1 (1.5)	2.6 (1.5)	1.3 (1.4)
CBI-R: “her/his appetite is greater, s/he eats more than before” (/4)	N/A	1.6 (1.4)	1.6 (1.3)	2.0 (1.7)
CBI-R: “table manners are declining e.g., stuffing food into mouth” (/4)	N/A	1.6 (1.5)	1.0 (1.1)	2.7 (1.8)

Values denote mean (standard deviation) or *n* (%)

*p* values denote significance on Mann–Whitney *U* or Chi square test

*N/A* not applicable, *FRS* frontotemporal dementia rating scale, *CBI-R* Cambridge Behavioural Inventory Revised version

* *p* < 0.05 disease group versus controls

### Imaging parameters

Volumetric T1- and T2-weighted MRI was performed in all 36 subjects. MRI scans were acquired on a 3T scanner (Tim Trio, Siemens) with the following sequences: (i) high-resolution isotropic 3D T1-weighted MPRAGE (sagittal orientation; TR = 2200 ms, TI = 900 ms, TE = 2.9 ms, flip angle = 10°, acquisition matrix = 256 × 256, and spatial resolution = 1.1 mm) and (ii) high-resolution isotropic 3D T2-weighted fast spin echo/SPACE (sagittal orientation; TR = 3200 ms, apparent TE = 105 ms, variable refocusing pulse flip angle to achieve T2-weighting, acquisition matrix = 256 × 256, and spatial resolution = 1.1 mm).

### Development of a hypothalamic segmentation protocol

A review of hypothalamic anatomy and previously described hypothalamic segmentation protocols was made [10, 12, 13, 17–23]. The most detailed segmentation protocol described was by Schindler et al. [23] which itself had been designed following a survey of previously published protocols. However, in that study, they used 7T T1-weighted MRIs which tend to be less widely available than 3T MRI. In order to optimize the protocol for 3T MRIs we made use of a volumetric T2-weighted MRI, acquired at the same time as the T1 image. By using the software package NiftyMIDAS (Centre for Medical Image Computing, UCL: http://cmic.cs.ucl.ac.uk/home/software/) which allows for the simultaneous viewing of different imaging modalities, we were able to perform a segmentation of the hypothalamus on registered volumetric T1- and T2-weighted images allowing better visualization of the boundaries of the hypothalamus (particularly laterally).

The segmentation protocol of Schindler et al. [23] was further optimized by reviewing descriptions of hypothalamic anatomy [17, 19–21] and criteria from other segmentation protocols [10, 12, 13, 18, 22]. Definitions of boundaries were made clearer, with greater detail provided in order to carefully include the hypothalamic nuclei in the segmentation (in particular the supraoptic, suprachiasmatic, retrochiasmatic nucleus, and the dorsal part of the arcuate (or infundibular) nucleus), and exclude the fornix. The protocol is defined in detail in the Supplementary Material.

### Methodology for segmentation

Acquired T1-weighted images were initially transformed into standard space by a rigid registration to the Montreal Neurological Institute (MNI305) template [23–26]. Acquired T2 images were registered to the MNI305 template, using a transformation which combines the “T2 to T1-native-space” and “T1 to MNI305-template” matrices (both derived after a six-parameter linear registration) using NiftyReg, revision #418 (Centre for Medical Image Computing, UCL: http://cmic.cs.ucl.ac.uk/home/software/). Segmentations were performed manually on coronal slices using NiftyMIDAS. Segmentations were first performed on the T1 image, and then edited, switching to the corresponding T2-weighted image which was superimposed on the T1.

### Reliability analysis

The reliability of this optimized segmentation protocol was tested in a sample of ten cognitively normal controls, scanned using the same MRI protocol as the study participants. Hypothalamic structures were segmented twice. The intraclass correlation (ICC) was computed with a two-way random effects model, with Dice overlapping coefficients computed using the Convert3D tool (www.itksnap.org/pmwiki/pmwiki.php?n=Convert3D.Convert3D). The intrarater absolute intraclass correlation coefficient (ICC) was 0.931 (95 % confidence intervals: 0.723–0.983) and the Dice values were 0.88 (standard deviation 0.02) for both right and left hypothalamic segmentations, confirming excellent reliability of the protocol.

### Development of a hypothalamic subsegmentation protocol

**Fig. 1:**
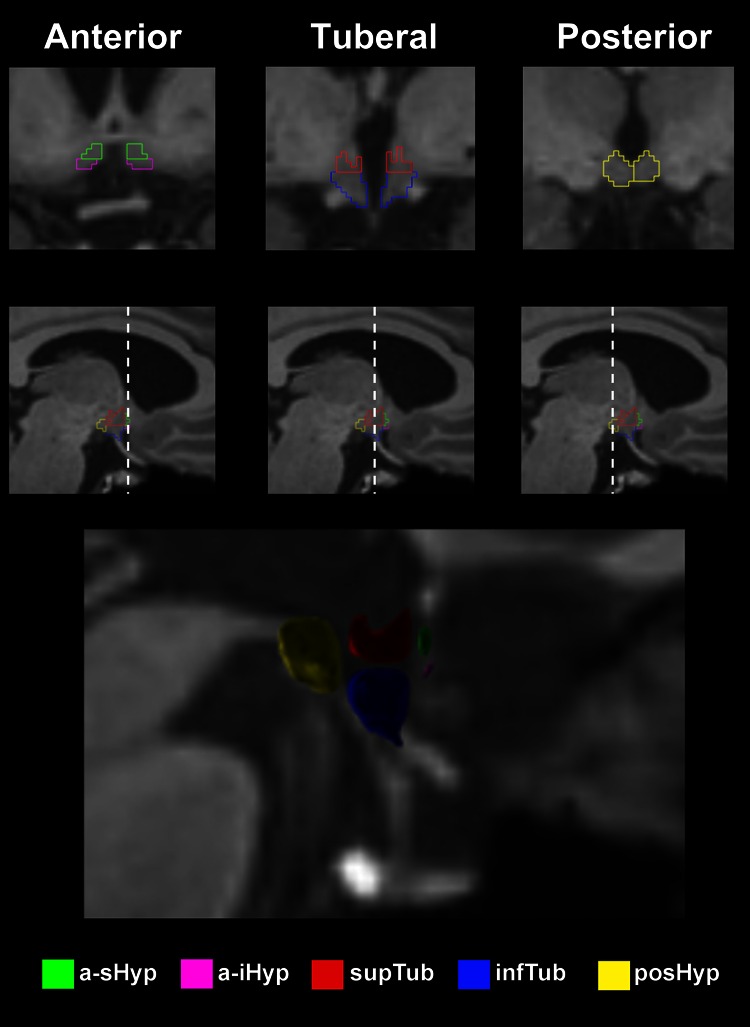
Segmentation of the hypothalamic subunits mapped on a 3T T1-weighted MR image of a control subject and their 3D reconstruction on a sagittal view. *a-sHyp* anterior superior hypothalamus, *a-iHyp* anterior inferior hypothalamus, *supTub* superior tuberal hypothalamus, *infTub* inferior tuberal hypothalamus, *posHyp* posterior hypothalamus

In order to investigate subregions of the hypothalamus in more detail, we adapted a methodology described by Makris et al. [21]. This uses visible anatomical landmarks on MRI scans to subsegment the hypothalamus into five subunits (Fig. 1): (i) the anterior superior hypothalamus (a-sHyp, which includes the paraventricular nucleus); (ii) the anterior inferior hypothalamus (a-iHyp, which includes the supraoptic nucleus); (iii) the superior tuberal hypothalamus (supTub, which includes the dorsomedial nucleus, the anterior part of the lateral hypothalamus, and the posterior part of the paraventricular nucleus); (iv) the inferior tuberal hypothalamus (infTub, which includes the arcuate (or infundibular) nucleus, the ventromedial nucleus and the posterior part of the supraoptic nucleus); and (v) the posterior hypothalamus (posHyp, which includes the posterior part of the lateral hypothalamus as well as the mammillary bodies). The detailed protocol for this subsegmentation is also included in the Supplementary Material.

### Methodology for subsegmentation

Using hypothalamic segmentations defined above, delineation of the hypothalamic subunits was also performed manually on coronal slices using NiftyMIDAS. This was performed initially on the T1 image, switching to the corresponding superimposed T2 image for editing.

### Calculation of hypothalamic volumes

Volumes of the whole hypothalamus and its subunits were automatically computed from the segmentations performed in NiftyMIDAS and corrected for total intracranial volume (TIV), which was calculated using the Statistical Parametric Mapping (SPM) 12b software, version 5953 (www.fil.ion.ucl.ac.uk/spm), running under Matlab R2012a (Math Works, Natick, MA, USA). The TIV corrected volume of a specific structure (i.e., the hypothalamus or a subunit) for each subject “i” was computed as follows: Structure volume_corrected(i)_ = Structure volume_raw(i)_*TIV_mean_/TIV_(i)_, where “Structure volume_raw(i)_” is the raw value of the structure of the subject “i,” “TIV_mean_” is the average TIV of the study group, and “TIV_(i)_” is the TIV of the subject “i.”

### Statistical analyses

Statistical analyses were performed in SPSS software (SPSS Inc., Chicago, IL, USA) version 12.0 and in R language v.3.0.2. Differences in demographic, cognitive, and clinical features as well as brain volumes were tested with the Mann–Whitney *U* test for continuous variables (due to the small sample size and to the non-normal distribution for all the variables) and Chi square test for dichotomous variables. For the hypothalamic subunit volumetry (five subunits on either side), a correction for multiple comparisons was made so that only a threshold of *p* < 0.005 was considered significant.

## Results

Demographic characteristics are reported in Table 1: no significant differences were found in gender, age, or education between controls and bvFTD, but the patient group scored significantly lower on the MMSE. All patients scored abnormally on the eating disturbance subscale of the CBI-R: 12 patients scored in the mild to moderate range (1–50 %) and 6 patients scored in the severe to very severe range (51–100 %).

No significant differences were seen in demographics, disease duration, MMSE, FRS, or total CBI-R score between the *MAPT* and *C9orf72* subgroups. Looking at the individual eating disturbance subscores of the CBI-R, there was a trend for higher scores in the *MAPT* group compared with *C9orf72* in wanting to eat the same foods repeatedly [mean (SD) score 2.6 (1.3) versus 1.3 (1.4)], with the opposite trend (higher score in *C9orf7*2) in decline in table manners [mean (SD) score 2.7 (1.8) versus 1.0 (1.1)].

The bvFTD group showed a 17 % lower total hypothalamic volume compared with controls (mean (SD) 783 (113) versus 944 (73) mm^3^, *p* < 0.0005, Mann–Whitney *U* test) with a similar reduction in both the right (17 %) and left (18 %) hypothalamus compared with controls. The *MAPT* mutation group showed a non-significant lower right and left hypothalamic volumes on both sides compared with *C9orf72* (10–13 % difference) (Table 2).

**Table 2:** Volumetry of hypothalamus and its subunits in 18 bvFTD (including nine *MAPT* and six *C9orf72* mutation carriers) and 18 control subjects

	Controls	bvFTD	% difference (negative means smaller in bvFTD than controls)	*MAPT* subgroup	*C9orf72* subgroup	% difference (negative means smaller in *MAPT* than *C9orf72*)
Hypothalamus—total	944 (73)	783 (113)**	−17	756 (133)**	854 (68)*	−11
Hypothalamus—right	477 (38)	398 (62)**	−17	380 (73)**	436 (41)*	−13
Hypothalamus—left	467 (39)	385 (53)**	−18	375 (63)**	418 (31)*	−10
Anterior superior—total	46 (18)	27 (13)**	−41	25 (13)**	25 (11)*	0
Anterior superior—right	22 (10)	13 (7)**	−43	12 (6)*	12 (7)*	0
Anterior superior—left	23 (9)	14 (6)**	−38	13 (7)*	14 (5)*	−7
Anterior inferior—total	30 (18)	18 (8)	−40	20 (8)	17 (8)	+18
Anterior inferior—right	15 (10)	9 (4)*	−40	8 (4)	8 (4)	0
Anterior inferior—left	15 (8)	10 (5)	−34	11 (5)	8 (5)	+38
Superior tuberal—total	289 (54)	225 (38)**	−22	213 (41)**	251 (30)	−15
Superior tuberal—right	145 (30)	114 (20)**	−21	108 (18)**^,^^	129 (19)	−16
Superior tuberal—left	144 (24)	111 (20)**	−23	105 (25)**	122 (12)*	−14
Inferior tuberal—total	317 (38)	314 (37)	−1	317 (48)	322 (22)	−2
Inferior tuberal—right	162 (20)	158 (20)	−2	158 (26)	163 (13)	−3
Inferior tuberal—left	155 (22)	156 (19)	+1	159 (23)	159 (11)	0
Posterior—total	263 (49)	199 (59)**	−24	181 (60)**	239 (49)	−24
Posterior—right	133 (27)	104 (34)*	−22	94 (35)**	124 (29)	−24
Posterior—left	130 (24)	95 (27)**	−26	87 (26)**	115 (26)	−24

Volumes are corrected for TIV. Values denote mean (standard deviation) volumes in mm^3^. *p* values denote significance on Mann–Whitney *U* test

* *p* < 0.05, ** *p* < 0.005 disease group versus controls; ^* p* < 0.05 *MAPT* versus *C9orf72* subgroups. Significance threshold was set at *p* < 0.005 to correct for multiple comparisons

The subsegmentation analysis revealed significant differences in the total (left and right combined) volumes of the superior regions (both anterior and tuberal) as well as the posterior region (superior tuberal 22 %, posterior 24 %, and anterior superior region 41 % smaller than controls), with a similar pattern when looking at the individual right and left volumes. No significant differences survived correction for multiple comparisons in the inferior regions (anterior and tuberal) between the bvFTD group and controls (Table 2).

Looking at the individual *MAPT* and *C9orf72* groups, only the *MAPT* mutation carriers showed significant differences from controls when corrected for multiple comparisons with superior (anterior and tuberal) and posterior regions being smaller. The *C9orf72* group showed a trend to smaller anterior superior (*p* = 0.009) and left superior tuberal regions (*p* = 0.047). Direct comparisons of the genetic groups revealed a trend for a smaller right superior tuberal region (16 %, *p* = 0.036) and posterior region (24 %, *p* = 0.066) in the *MAPT* group compared with *C9orf72*.

In the total bvFTD group, patients who scored in the severe to very severe range of the CBI-R eating disturbance subscale had a trend to a lower total hypothalamic volume [740 (89) mm^3^] than those in the mild to moderate range [805 (122) mm^3^]. This trend for a lower volume in those scoring in the severe to very severe range was also seen in the superior tuberal region [205 (36) versus 236 (37) mm^3^]. Similarly, in both genetic groups, there was a non-significantly lower total hypothalamic volume in those scoring within the severe to very severe range: in the *MAPT* group 721 (120) versus 773 (147) mm^3^, in the *C9orf72* group 794 (35) versus 884 (60) mm^3^.

## Discussion

Using a novel segmentation protocol for the hypothalamus and its subunits based upon registered volumetric T1 and T2 MR images, we have shown that the hypothalamus is substantially smaller in patients with bvFTD compared with controls, particularly in the superior and posterior regions. There was also a trend for a smaller hypothalamus, particularly in the superior tuberal region, in those who had severe eating disturbance. Furthermore, there is significant atrophy in the *MAPT* mutation group (in superior and posterior areas), but no significant differences from controls in the *C9orf72* mutation group.

Our findings are different from the only previous study of hypothalamic volume in FTD [10], which found significant atrophy in the posterior hypothalamus. Our study differed from this in a number of aspects, both technical and clinical. Technically, we used different criteria for the segmentation of the hypothalamus, in particular, paying attention to the exclusion of the fornix, and inclusion of the supraoptic, suprachiasmatic, retrochiasmatic, and arcuate (or infundibular) nuclei. Furthermore, we were able to delineate five specific subunits of the hypothalamus, whereas Piguet et al. [10] used an arbitrary definition of anterior and posterior regions of the hypothalamus, splitting it through the middle coronal plane of their segmentation. Their ‘posterior’ hypothalamus may therefore include part of the tuberal region as we have defined it in this study. Clinically, it is unclear whether the cohorts overlap as their 18 patients are not genetically defined unlike the group studied here which contains a significant number of genetic bvFTD cases. The Piguet cohort has an earlier mean disease duration (3.3 years versus 9.1 years here), although disease severity is similar (MMSE 23.9 versus 25.0 here; CBI-R eating disturbance score 6.9 versus 7.7 here). In a separate pathological analysis, they investigated six tau-positive and six TDP-43 positive FTD cases, also finding problems more posteriorly, attributing this to atrophy in the TDP-43 group. However, their tau group contained only cases with a specific type of tau pathology, Pick’s disease (a 3-repeat tauopathy), and their TDP-43 group similarly contained only cases with one subtype (TDP-43 type B). Recent studies have made it clear that there are large differences both clinically and pathologically between the different tau and TDP-43 pathological subtypes (which number at least four in each group), and so one cannot extrapolate to a significant difference between ‘all TDP-43’ and ‘all tau’ pathology by investigating only one subtype. Our study suggests that a particular tau group (*MAPT* mutations) appear to have significant hypothalamic volume loss compared with a TDP-43 group (*C9orf72* expansions), the opposite finding to Piguet et al. [10].

We found that patients with more severe eating disturbance had a trend to lower hypothalamic volumes, particularly within the superior tuberal region. However, the differences did not reach statistical significance and so caution should be attributed to these findings until further studies are performed. The CBI-R provides only limited information on the many different types of abnormal eating behaviors, as only four questions are asked. Eating behaviors in bvFTD are complex and varied, and include different aspects, such as carbohydrate craving, overeating, obsessions for specific foods and oral exploration of inedible objects, which may not always coexist in an individual patient [7, 27, 28]. There was a trend for a difference in the type of eating behaviors exhibited between the two genetic groups with the *MAPT* group scoring higher on wanting to eat the same foods repeatedly and the C*9orf72* group showing a greater decline in table manners. Further studies are required using more detailed feeding questionnaires to explore these issues further [7].

This study found significant atrophy in the superior and posterior subunits of the hypothalamus which contain the paraventricular nucleus (anterior superior region), dorsomedial nucleus (superior tuberal region), and lateral hypothalamic areas (superior tuberal and posterior regions). These subnuclei are all involved in important aspects of appetite regulation and contain neuropeptide-expressing neurons and neuropeptide receptors [20, 29–32]. Interestingly, the inferior tuberal area was not atrophic, an area which contains the arcuate (or infundibular) and ventromedial nuclei. The arcuate nucleus is the primary target of metabolic and hormonal signals from the periphery, with important connections to other nuclei in the hypothalamus particularly the paraventricular nucleus [30]. This suggests that appetite abnormalities in bvFTD could be due to changes in neuropeptides (or neuropeptide receptors) within the superior and posterior areas of the hypothalamus and/or from disruption of pathways from the arcuate nucleus to other areas of the hypothalamus. As there is differential neuropeptide expression within these nuclei, different neuropeptide levels should be impaired compared with others in bvFTD: such a hypothesis has yet to be explored.

There was a trend for greater posterior involvement in the *MAPT* group compared with the *C9orf72* group, with both left and right posterior regions 24 % smaller in the *MAPT* group. As well as the posterior part of the lateral hypothalamus, the posterior region contains the mammillary bodies, which are connected to the amygdala and hippocampus, areas known to be major areas of atrophy in patients with *MAPT* mutations [33]. Given this finding, future studies should further investigate the relationship of memory impairment in these patients to hypothalamic atrophy.

In summary, bvFTD patients had smaller hypothalamic volumes compared with controls, with atrophy localized to subnuclei regulating food intake, reward and perception of satiety, and related to the severity of the eating disturbance. Moreover, different genetic mutations seem to have a differential impact on the hypothalamus, although further studies in larger genetic and pathological series are required to confirm this. The structural and functional connections of the hypothalamus should be further explored in bvFTD, particularly how they relate to the orbitofrontal-insular-striatal reward network previously identified. Lastly, the results of this study suggest testable hypotheses of the role of different neuropeptides in impaired appetite regulation in bvFTD.

## Electronic supplementary material

Supplementary material 1 (PDF 1275 kb)
