# Supplementary material for: Detailed volumetric analysis of the hypothalamus in behavioral variant frontotemporal dementia
Source: J Neurol. 2015 Sep 4;262(12):2635–42. doi: 10.1007/s00415-015-7885-2 (PMC4655011; doi:10.1007/s00415-015-7885-2)
Supplement: Supplementary file 1 — Supplementary material 1 (PDF 1275 kb) [file 415_2015_7885_MOESM1_ESM.pdf]

SUPPLEMENTARY MATERIAL

Criteria for the manual segmentation of the hypothalamus and its subunits on 3T MRI

1. Introduction

This protocol describes how to manually segment the human hypothalamus on magnetic resonance images (MRIs), combining the information obtained on volumetric T1- and T2-weighted images (Figure e-1, Sections 2 and 3). It also includes a description of how to subsequently segment five hypothalamic subregions, using an adaptation of the criteria reported in Makris et al., [22] (Figure e-1).

2. Segmentation procedures

2.1 Image Orientation/Registration/Standard space

The segmentations are made on MRIs rigidly registered to Montreal Neurological Institute (MNI) standard space. T2-weighted images are co-registered to the T1 images.

2.2 Direction of segmentation

Segmentation proceeds on contiguous coronal slices in the rostral-caudal direction. For a slice thickness of 1 mm, approximately 12-15 slices include the hypothalamus (Figure e-1).

2.3 3D navigation

For some landmarks, especially the fornix and the arcuate (or infundibular) nuclei (Figures e-2 and e-3), the morphological details visible in the coronal slice are not sufficient to determine whether the tissue belongs to the hypothalamus or not. To perform accurate segmentation, the axial and sagittal planes must be checked as aids, using visualization tools allowing 3D navigation.

3. Segmentation landmarks

The segmentation includes the preoptic area, the anterior hypothalamic area, the tuberal and the mammillary region.

3.1 Most rostral slice

The hypothalamic segmentation begins on the very first slice where the anterior commissure appears continuous (Figure e-1A). In many brains this should also correspond to the first slice on which the optic tract is attached to the brain by two 'wisps' of white matter [10].

3.2 Most caudal slice

The hypothalamus ends at the slice on which the caudal edge of the mammillary bodies is still clearly visible (Figure e-1N). This definition tolerates a potential loss of the posterior tips of the lateral hypothalamic area and posterior hypothalamic area, which can extend beyond the mammillary bodies.

3.3 Ventral boundary

The hypothalamus borders with the medial ventral part of the floor of the diencephalon. Rostrally it rests dorsal to the optic chiasm and then it is encircled by the caudally emerging optic tracts. The supraoptic, suprachiasmatic and retrochiasmatic nucleus must be carefully included in the segmentation. The supraoptic nucleus caps the optic chiasm and straddles the optic tract laterally [21]. The suprachiasmatic nucleus is small and situated above the optic chiasm, close to the ependymal lining of the third ventricle [12]. Rostro-laterally, it blends into the chiasmatic grey, and caudally it gradually merges with the retrochiasmatic nucleus. This latter, also called the anterior hypothalamic nucleus, is localized dorsally, between and ventrally the fibers of the supraoptic commissures [12]. Just caudal to the optic chiasm, the retrochiasmatic nucleus borders ventro-laterally upon the arcuate (or infundibular) nucleus of the tuberal region, which extends into the median eminence and the hypothalamic tuber cinereum, the bulb from which the infundibulum extends to the hypophysis [12] (Figure e-2).

3.4 Dorsal boundary

In the most rostral slices, the dorsal border is defined by the anterior commissure, while for the remaining caudal slices the hypothalamic sulcus (or sulcus of Monro) serves as the major dorsal boundary of the hypothalamus caudal to the anterior commissure. This is a suitable landmark for most substructures of the hypothalamus, with the exception of the paraventricular nucleus, which can extend beyond this line. In addition, the dorsal end of the fornix, the posterior limb of the internal capsule, and the mammillo-thalamic tract can be used as landmarks [13, 22]. The anatomical dorsal boundary of the medio-caudal hypothalamus adjoins the ventral thalamus which has similar intensity in T1-weighted images.

3.5 Medial boundary

Surrounded rostrally and ventrally by the hypothalamus, the third ventricle is the prominent landmark to define the medial boundary and the interhemispheric fissure can be used to separate the right from the left hypothalamus. Attention should be taken in including the paraventricular nucleus of the hypothalamus: it forms a vertical plate of grey matter immediately beneath the ependyma of the third ventricle (Figure e-1D-G, label 12). Ventral to the fornix, the main body of the paraventricular nucleus rises vertically along the wall of the third ventricle throughout the preoptic and tuberal regions of the hypothalamus [12, 21].

3.6 Lateral boundary

The anatomical landmarks of the lateral boundary are the white matter of the internal capsule (bordering the globus pallidus) and the cerebral peduncle. The lateral hypothalamic area adjoins non-hypothalamic grey matter structures, for example the substantia innominata (including the basal nucleus of Meynert), the great terminal island (or islands of Calleja), the substantia nigra, the subthalamic nucleus and the bed nucleus of the stria terminalis [13]. The lateral boundary of the hypothalamus is difficult to detect on T1 images alone due to the intensity transition area between the hypothalamus and the intermingled white matter fibres (the diagonal band of Broca, the medial forebrain bundle, the sublenticular stria and the ventral amygdalofugal pathway), as also reported by Gabery et al. [18]. However, the combined use of information from T1 and T2-weighted images allows reliable exclusion of the internal capsule and the diagonal band of Broca from the lateral boundary of the hypothalamus, and the zona incerta and the fields of Forel H2 on the most caudal slices, structures which are difficult to discern on a T1 image alone.

The lateral edge of the optic tract (as the optic chiasm bifurcates bilaterally) can be used as a landmark for the lateral border of the preoptic hypothalamus [13, 22]. In those slices where there are no visible morphological details, an arbitrary straight line can be drawn from the most lateral edge of the optic tract to the fornix, to the internal capsule (rostrally), or to the hypothalamic sulcus (caudally) [10].

3.7 Exclusion of the fornix

The white matter of the fornix is excluded from the segmentation, even when surrounded by the grey matter of the hypothalamus (i.e. the column of the fornix caudal to the preoptic area) (Figure e-1E-J). The sagittal view needs to be checked as an aid for the exclusion of the fornix (Figure e-3). [Note that the white matter within the

hypothalamic grey matter is included as it belongs to the pallidohypothalamic fibres, the mammillary body, or the junction of the mammillary body with the mammillary fasciculus.]

4. Segmentation of hypothalamic subunits

Once segmented as a whole, the hypothalamus can be subdivided into five subunits as defined by Makris et al. [22] (Figure e-1):

1. anterior superior (a-sHyp)
2. anterior inferior (a-iHyp)
3. superior tuberal (supTub)
4. inferior tuberal (infTub)
5. posterior (posHyp)

The lateral and medial boundaries of each subunit correspond to the boundary of the whole nucleus, thus the more fine-grained subdivision only requires definition of their rostro–caudal and dorso–ventral borders.

The anterior hypothalamic subunits (i.e. a-sHyp and a-iHyp) extend from the first rostral slice where the hypothalamus is segmented to the rostral most tip of the infundibulum. The tuberal subunits (i.e. supTub and infTub) extend from the rostral most section containing the infundibulum to the coronal slice just rostral to the one where the mammillary body is clearly visible. The posHyp subunit is segmented from the first rostral slice where the mammillary body is clearly visible to the most caudal one where the whole hypothalamus is detected, as described above.

The border between the a-sHyp and a-iHyp subunits, as well as between the supTub and infTub subunits, was set at the dorsal most level of the floor of the substantia innominata or the anterior and lateral perforated substance.

Figure e-1. Example of hypothalamus segmentation on the ICBM152 2009c Nonlinear Symmetric - 1x1x1mm template (McConnell Brain Imaging Centre, Montreal Neurological Institute, McGill University). All 1 mm slices are shown, in the rostro-caudal direction.

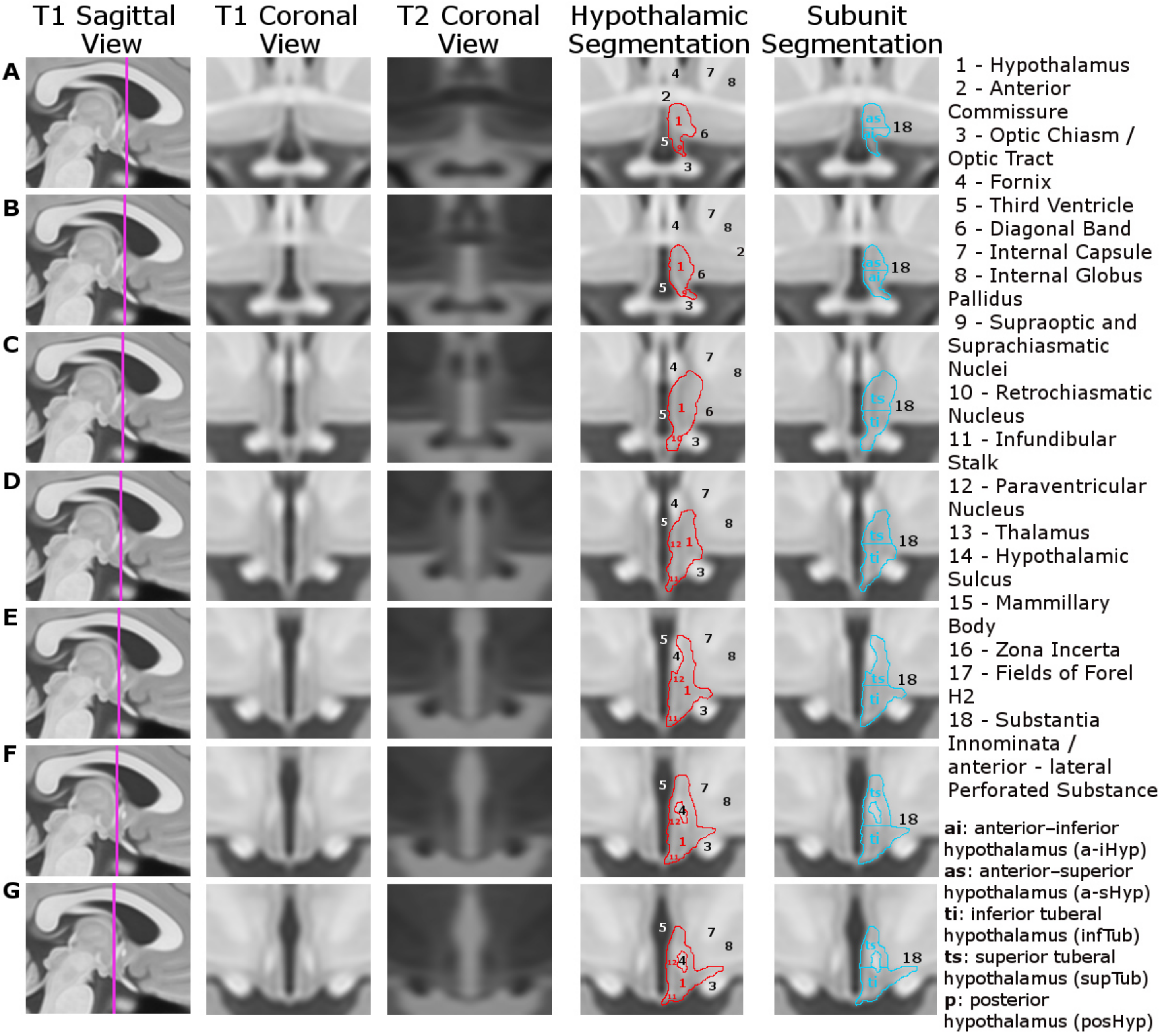

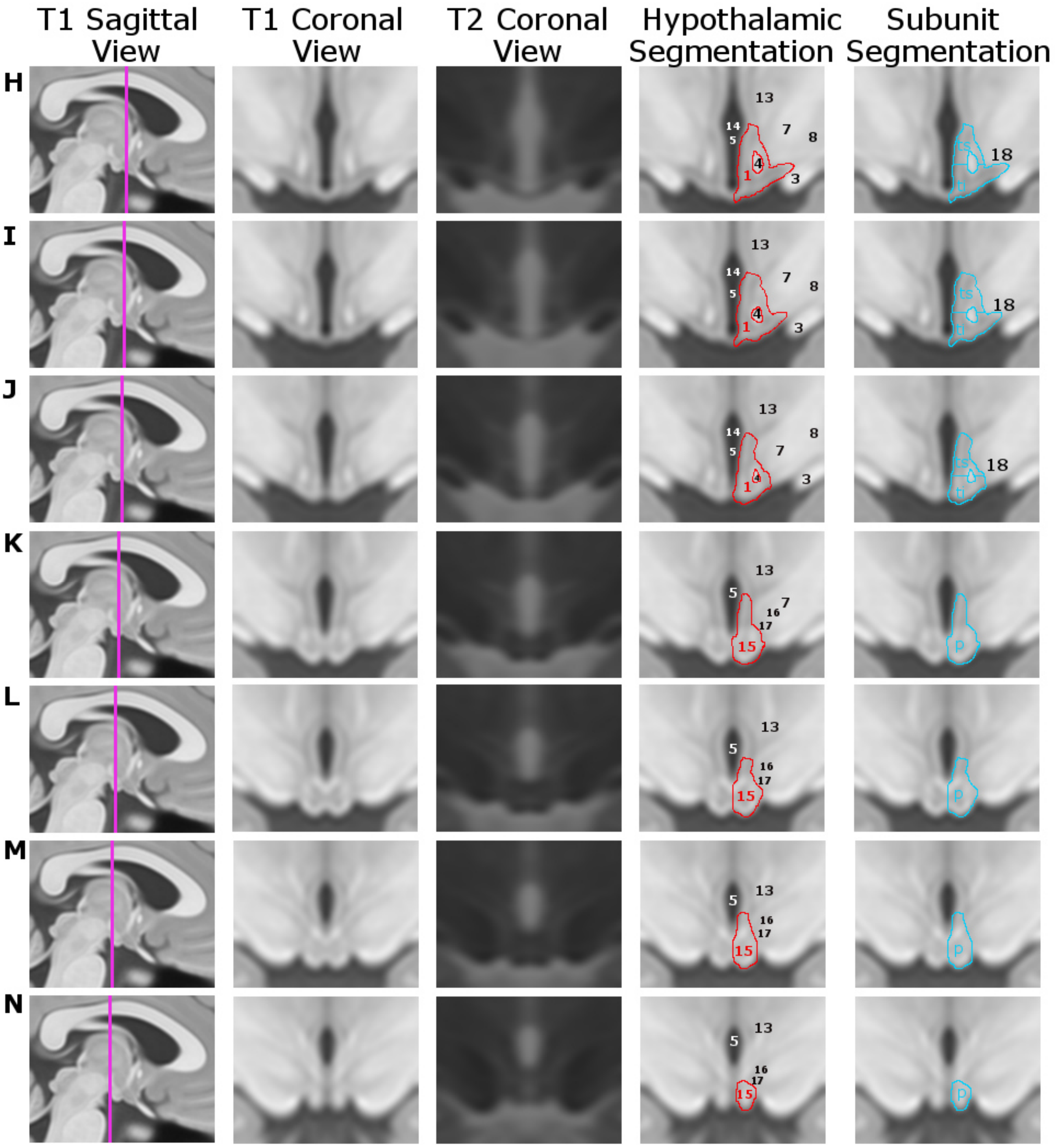

1 - Hypothalamus
2 - Anterior Commissure
3 - Optic Chiasm / Optic Tract
4 - Fornix
5 - Third Ventricle
6 - Diagonal Band
7 - Internal Capsule
8 - Internal Globus Pallidus
9 - Supraoptic and Suprachiasmatic Nuclei
10 - Retrochiasmatic Nucleus
11 - Infundibular Stalk
12 - Paraventricular Nucleus
13 - Thalamus
14 - Hypothalamic Sulcus
15 - Mammillary Body
16 - Zona Incerta
17 - Fields of Forel H2
18 - Substantia Innominata / anterior - lateral Perforated Substance

ai: anterior–inferior hypothalamus (a-iHyp)
as: anterior–superior hypothalamus (a-sHyp)
ti: inferior tuberal hypothalamus (infTub)
ts: superior tuberal hypothalamus (supTub)
p: posterior hypothalamus (posHyp)

Figure e-2. Identification of the arcuate (or infundibular) nucleus, the optic tract on sagittal and coronal views using the ICBM152 2009c Nonlinear Symmetric - 1x1x1mm template (McConnell Brain Imaging Centre, Montreal Neurological Institute, McGill University).

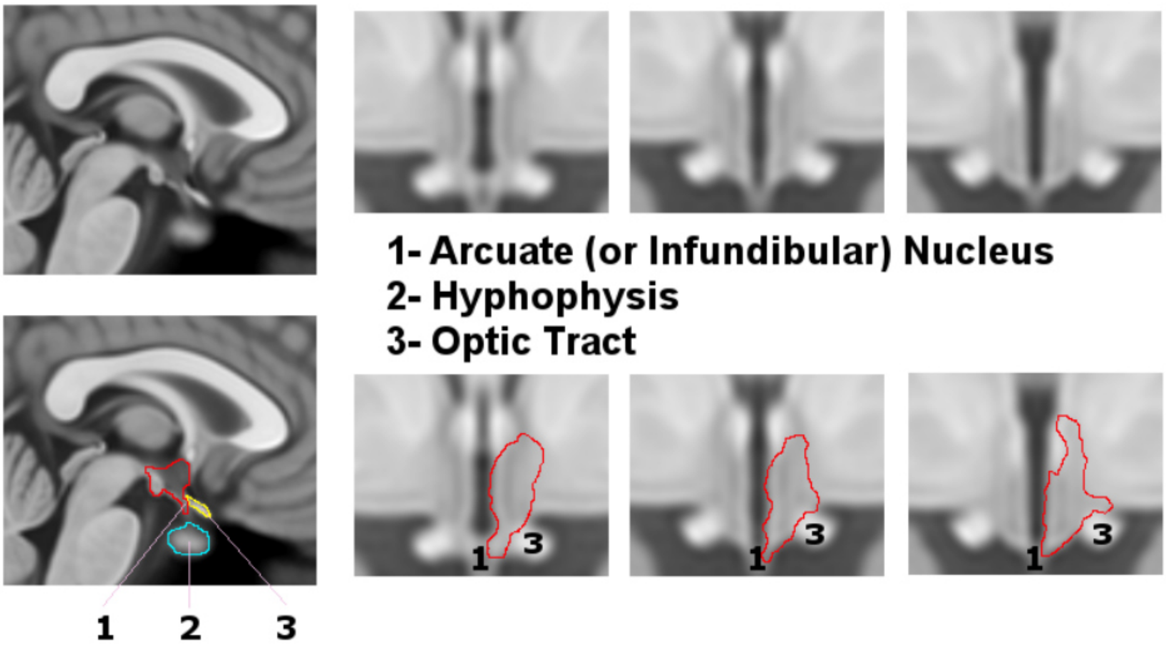

Figure e-3. Identification of the fornix to be excluded from the hypothalamic segmentation on sagittal and coronal views using the ICBM152 2009c Nonlinear Symmetric - 1x1x1mm template (McConnell Brain Imaging Centre, Montreal Neurological Institute, McGill University).

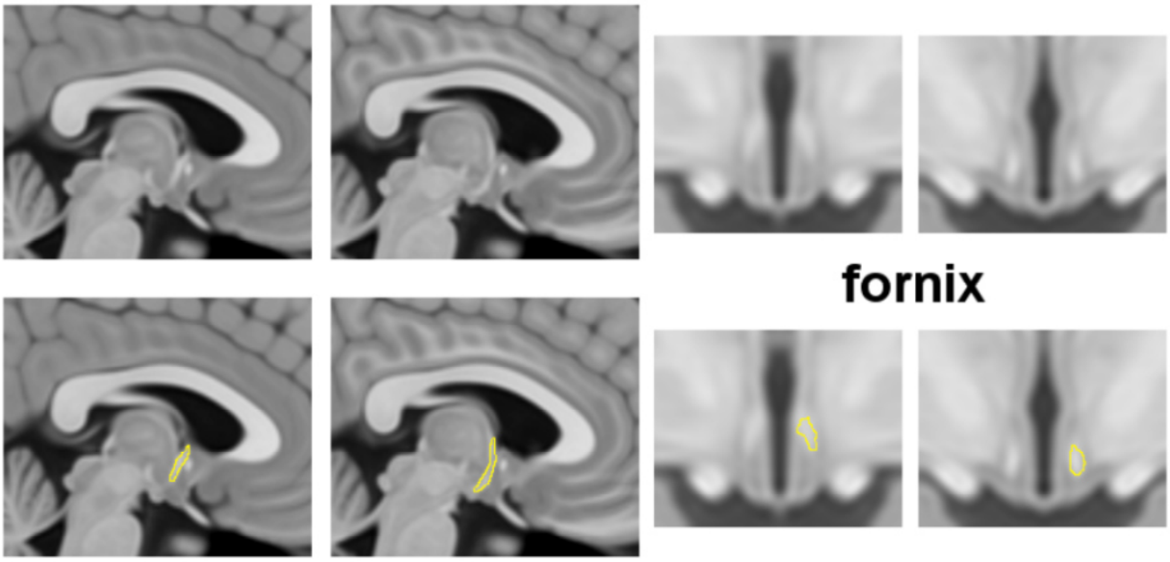

Table e-1. Summary of landmarks of the hypothalamic segmentation protocol. This table provides an overview of landmarks, adapted from [23]. Coronal segmentation of hypothalamic grey matter: start at first slice with continuous anterior commissure and continue for each slice to the caudal end of the mammillary body (MB); preferentially select landmarks in order of hierarchy as described below ('>'); in case of doubt select the landmark which can be best identified; landmarks divided by a comma (',') are of the same hierarchical level; * include in segmentation.

**1. Coronal landmarks**	**Ventral**	**Dorsal**	**Medial**	**Lateral**
**Preoptic area**<br>(first slice on which the anterior commissure appears continuous); (Figure e-1A)	Optic chiasm	Anterior commissure, column of the fornix	Midline between hypothalami, third ventricle, lamina terminalis	Diagonal band > medial forebrain bundle > lateral edge of the optic chiasm
**Anterior hypothalamic area**<br>(first slice with ipsilateral inter-ventricular foramen); (Figure e-1B to G)	Cerebral exterior, infundibular stalk*	1st slice: ventral to fornix, 2nd slice: lateral to fornix, 3rd slice: dorsal to fornix > medial pole of the internal capsule	Midline between hypothalami, third ventricle	Internal capsule, internal globus pallidus, max. lateral edge of the optic tract
**Tuberal region**<br>(anterior pole of ipsilateral anteroventral/ -medial thalamus); (Figure e-1H to J)	Cerebral exterior	Hypothalamic sulcus > medial pole of the internal capsule > intersection of ventricle wall and extended dorsal edge of putamen	Midline between hypothalami, third ventricle	Internal capsule, internal globus pallidus
**Mammillary region**<br>(rostral and caudal extent of ipsilateral MB);	Cerebral exterior	Hypothalamic sulcus > inferior thalamic peduncle > medial pole of field H2 >	Midline between MBs, third ventricle	Zona incerta, cerebral peduncle, substantia nigra,

(Figure e-1K to N)		mammillo-thalamic tract > mammillary fasciculus (incl. convergence with MB) > anterior commissure		subthalamic nucleus, mammillo-thalamic tract
**2. Axial landmarks**	**Rostral**	**Lateral**	**Medial**	**Caudal**
**Transverse level of anterior commissure**	Anterior commissure	Stria medullaris of thalamus, inferior thalamic peduncle	Midline between hypothalami, third ventricle	Thalamus >mammillo-thalamic tract
**Transverse level of diagonal band**	Diagonal band	Diagonal band, stria medullaris of thalamus, inferior thalamic peduncle, cerebral peduncle, ansa lenticularis, mammillo-tegmental tract	Midline between hypothalami, third ventricle, lamina terminalis	End of MB > posterior margin of mammillo-thalamic tract
**Transverse level of optic tract**	Cerebral exterior	Optic tract, ansa lenticularis	Third ventricle, Midline in sagittal plane between MBs	End of MB, cerebral peduncle
**3. Sagittal landmarks**	**Rostral**	**Dorsal**	**Ventral**	**Caudal**
**Medial – lateral**	Diagonal band	Hypothalamic sulcus, inferior thalamic peduncle, thalamus	Cerebral exterior, hyphophysis	field H2, mammillary fasciculus, zona incerta
